# Verification of *TRI3* Acetylation of Trichodermol to Trichodermin in the Plant Endophyte *Trichoderma taxi*

**DOI:** 10.3389/fmicb.2021.731425

**Published:** 2021-10-25

**Authors:** Haijiang Chen, Lijuan Mao, Nan Zhao, Chenyang Xia, Jian Liu, Christian P. Kubicek, Wenneng Wu, Su Xu, Chulong Zhang

**Affiliations:** ^1^College of Food and Pharmaceutical Engineering, Guiyang University, Guiyang, China; ^2^Institute of Biotechnology, Zhejiang University, Hangzhou, China; ^3^Technology Center, China Tobacco Guizhou Industrial Co., Ltd., Guiyang, China; ^4^Analysis Center of Agrobiology and Environmental Science, Zhejiang University, Hangzhou, China; ^5^Microbiology Group, Research Area Biochemical Technology, Institute of Chemical, Environmental and Biological Engineering, TU Wien, Vienna, Austria

**Keywords:** *Trichoderma*, *TRI3*, trichodermin biosynthesis, antifungal activity, trichothecene

## Abstract

Trichodermin, a trichothecene first isolated in *Trichoderma* species, is a sesquiterpenoid antibiotic that exhibits significant inhibitory activity to the growth of many pathogenic fungi such as *Candida albicans*, *Rhizoctonia solani*, and *Botrytis cinerea* by inhibiting the peptidyl transferase involved in eukaryotic protein synthesis. Trichodermin has also been shown to selectively induce cell apoptosis in several cancer cell lines and thus can act as a potential lead compound for developing anticancer therapeutics. The biosynthetic pathway of trichodermin in *Trichoderma* has been identified, and most of the involved genes have been functionally characterized. An exception is *TRI3*, which encodes a putative acetyltransferase. Here, we report the identification of a gene cluster that contains seven genes expectedly involved in trichodermin biosynthesis (*TRI3*, *TRI4*, *TRI6*, *TRI10*, *TRI11*, *TRI12*, and *TRI14*) in the trichodermin-producing endophytic fungus *Trichoderma taxi*. As in *Trichoderma brevicompactum*, *TRI5* is not included in the cluster. Functional analysis provides evidence that TRI3 acetylates trichodermol, the immediate precursor, to trichodermin. Disruption of *TRI3* gene eliminated the inhibition to *R. solani* by *T. taxi* culture filtrates and significantly reduced the production of trichodermin but not of trichodermol. Both the inhibitory activity and the trichodermin production were restored when native *TRI3* gene was reintroduced into the disruption mutant. Furthermore, a His-tag-purified TRI3 protein, expressed in *Escherichia coli*, was able to convert trichodermol to trichodermin in the presence of acetyl-CoA. The disruption of *TRI3* also resulted in lowered expression of both the upstream biosynthesis *TRI* genes and the regulator genes. Our data demonstrate that *T. taxi TRI3* encodes an acetyltransferase that catalyzes the esterification of the C-4 oxygen atom on trichodermol and thus plays an essential role in trichodermin biosynthesis in this fungus.

## Introduction

Trichothecenes are a group of naturally occurring sesquiterpenoids capable of inhibiting the function of eukaryotic ribosomes, thereby causing hemorrhagic lesions, dermatitis, and immunological problems to people and animals (Pace et al., 1998; McLaughlin et al., 2009; McCormick et al., 2011). All trichothecenes contain a core tricyclic 12,13-epoxytrichothec-9-ene (EPT) structure with an epoxide substituent and could be divided into four different types according to the pattern of esterification and oxygenation (Ueno, 1984; Ueno and Hsieh, 1985; Cole et al., 2003). Trichodermin belongs to the type that lacks the oxygen substitution at C-8 while containing a hydroxyl residue at C-4 and is renowned as a very potent inhibitor of protein synthesis in eukaryotic cells (Carrasco et al., 1973; Cundliffe and Davies, 1977; Kubicek and Druzhinina, 2015). It primarily interferes in the chain-termination process on the ribosome by blocking the interaction of the peptidyl transferase with the peptide release factor (Wei et al., 1974). Trichodermin was first isolated from a *Trichoderma* species as an antifungal antibiotic that is active against a variety of pathogenic fungi including *Candida albicans* (Godtfredsen and Vangedal, 1965). This sesquiterpenoid metabolite is known to be produced by only two *Trichoderma* species (*Trichoderma brevicompactum* and *Trichoderma arundinaceum*; Kubicek and Druzhinina, 2015; Nielsen et al., 2005) and exhibits significant inhibition to several human pathogenic *Candida* spp. and a nosocomial strain of the filamentous fungus *Aspergillus fumigatus* (Tijerino et al., 2011). The ability of these *Trichoderma* species to produce trichodermin, to secrete extracellular hydrolytic enzymes, and to directly interact with plants has made them effective biocontrol agents against plant pathogens like *Rhizoctonia solani*, *Botrytis cinerea*, and some soil pathogens (e.g., Fusarium spp.; Howell, 2003; Kulikov et al., 2006; Malmierca et al., 2012). A silkworm infection assay demonstrated that trichodermin yielded therapeutic effects on silkworms infected by *C. albicans* (Uchida et al., 2016). Recently, trichodermin has drawn attentions from cancer biologists because it can induce cell apoptosis through mitochondrial dysfunction and endoplasmic reticulum stress in human chondrosarcoma cells and by mitotic arrest and DNA damage in human p53-mutated pancreatic cancer cells (Su et al., 2013; Chien et al., 2016). These new findings suggest that trichodermin is a potential therapeutic agent worthy of further development to a clinical trial candidate for treating cancer. To this end, a thorough understanding of the biosynthetic pathway leading to trichodermin is essential.

The study of trichothecene biosynthesis has been pioneered in *Fusarium* spp. In *Fusarium*, most of *TRI* genes are located in the so-called *TRI5* cluster, which consists of *TRI3*, *TRI4*, *TRI5*, *TRI6*, *TRI8*, *TRI9*, *TRI10*, *TRI11*, *TRI12* and *TRI13* (Brown et al., 2004). Three other genes occur at separate distant positions: *TRI101* and *TRI1*–*TRI16* cluster, which are physically close (Kimura et al., 2007). Thereby, *TRI6* and *TRI10* genes encode transcriptional regulators (Tag et al., 2001; Kimura et al., 2007; Nasmith et al., 2011), and *TRI12* encodes a transport protein for export of trichothecenes (Alexander et al., 1999), while the remaining genes in *TRI5* cluster encode enzymes involved in trichothecene biosynthesis (Alexander et al., 2004; Kimura et al., 2007).

Trichothecene biosynthesis in *Trichoderma* shares several features with that in *Fusarium* yet exhibits some distinct differences in gene clustering, gene arrangement, and gene functions (Malmierca et al., 2013). For example, orientation of part of *TRI* genes is opposite to that in *Fusarium* (Cardoza et al., 2011), and *TRI5* gene—which encodes a trichodiene synthase that in *T. arundinaceum* coverts farnesyl pyrophosphate (FPP) into trichodiene, consistent with the function of *Fs*TRI5 in trichothecenes biosynthesis (Hohn and Middlesworth, 1986)—is not present in the *TRI* cluster (Cardoza et al., 2011). In addition, some enzymes encoded by the *TRI* cluster show several catalytic differences: the cytochrome P450 monooxygenase *Ta*TRI4 exhibits a different pattern of oxidation: in *T. arundinaceum*, *Ta*TRI4 introduces oxygen into trichodiene at positions C-2, C-11, and C-13 (Cardoza et al., 2011), whereas the *Fusarium* orthologue TRI4 further targets position C-3 (McCormick et al., 2006; Tokai et al., 2007); *Ta*TRI11 of *T. arundinaceum* functions as a hydroxylase, which catalyzes the hydroxylation of EPT at the C-4 position in contrast to that of the C-15 position in *Fusarium sporotrichioides* (Alexander et al., 1998). TRI3 of *Fusarium* is responsible for acetylation of hydroxyl group at C-15 in T-2 toxin biosynthesis (McCormick et al., 1996; Cardoza et al., 2011), while the role of *TRI3* in *Trichoderma* has not been characterized until recently. TRI3 had been proposed to take part in the acetylation of C-4 hydroxyl on trichodermol in *Trichoderma* (Cardoza et al., 2011) and was evidenced to be responsible for trichodermin biosynthesis in an endophytic *T. brevicompactum* by mutational analysis (Shentu et al., 2018). Yet evidence showing direct participation of TRI3 in trichodermol biotransformation is still lacking, and study of trichodermin biosynthetic pathway in other *Trichoderma* species rather than *T. arundinaceum* and *T. brevicompactum* has not been reported.

We have previously identified a novel trichodermin-producing endophytic fungus from Chinese yew *Taxus mairei*, *Trichoderma taxi* ZJUF0986 (Zhang et al., 2007). It was then shown to inhibit the growth of several fungal plant pathogens, including (but not limited to) *B. cinerea* and *R. solani* (Zhang et al., 2007; Chen et al., 2008; Shentu et al., 2013). The objective of this study was thus to identify the core genes responsible for the synthesis of trichodermin in the species and—more importantly—to investigate the role of *TRI3* in producing trichodermin in *Trichoderma*. The results reveal that *TRI* clusters in *T*. *taxi* are significantly similar to those in *T. arundinaceum* and *T. brevicompactum* and that *TtTRI3* encodes an acetyltransferase that performs the step by acetylating trichodermol at C-4 to trichodermin.

## Materials and Methods

### Microbial Strains Used

Endophytic *T. taxi* ZJUF0986 was isolated from *T. mairei* (Zhang et al., 2007) and is available in the China Center for Type Culture Collection under CGMCC No. 1672. *R. solani* (ACCC 3614) was used as a test pathogen as described previously (Wang et al., 2012). *T. taxi* and its derivatives and *R. solani* were maintained on potato dextrose agar (PDA) plates at 25°C in the dark. *Escherichia coli* DH5α was used for regular cloning and plasmid maintenance. *E. coli* M15 (Qiagen, Hilden, Germany) was adopted as protein expression host.

### Identification and Sequence Analysis of *TRI* Gene Cluster in *Trichoderma taxi*

The genome of ZJUF0986 was obtained by sequencing (manuscript in preparation). Nucleotide and amino acid sequences of *TRI* genes from *T. arundinaceum* and *T. brevicompactum* were retrieved from National Center for Biotechnology Information (NCBI). Initially, *TaTRI3* gene was used for BLASTn search against ZJUF0986 genome to identify the contig containing the *TRI3* orthologue in ZJUF0986. The contig was then used as a query to search for other *TRI* genes by BLASTx.^1^ The organization of the encoded open reading frames (ORFs) was illustrated by pDRAW32.^2^ Alignment of the amino sequences of all the putative *T. taxi* TRI proteins with those from *T. arundinaceum* and *T. brevicompactum* was performed by MEGA5.0 (Tamura et al., 2011) and then reviewed by Genedoc (Nicholas et al., 1997). All TRI amino sequences were subjected to protein domain analysis by Pfam^3^ (Finn et al., 2014).

### Generation of *TtTRI3* Gene Knockout Mutants and Complementation

The gene deletion vector was constructed based on the double-joint PCR strategy (Yu et al., 2004). Briefly, approximate 1 kb upstream and 1 kb downstream sequences flanking *TtTRI3* locus were amplified with the primers TRI3-upF/R and TRI3-downF/R, respectively. A 1.4-kb hygromycin B resistance (*HPH*) cassette was cloned from pCB1003 (Carrol et al., 1994) with primers HPH-F/R. The three fragments were mixed together in the second-round PCR, the product of which acted as template for the final amplification with nested primers TRI3-NestF/R. The double-jointed PCR product was purified and subcloned into the commercial TA-cloning vector pMD18-T (TaKaRa, Dalian, China). The resulting plasmid and the *Agrobacterium* binary vector for plant transformation, pCAMBIA1300 (GenBank: AF234296; Hajdukiewicz et al., 1994), were both cut with *Hin*dIII/*Xba*I and then joined together by T4 DNA ligase (TaKaRa), leading to the final *TtTRI3* gene knockout vector, pCAMBIA-TRI3. To obtain the *TtTRI3* null mutants, pCAMBIA-TRI3 was transformed into the wild strain using *Agrobacterium tumefaciens*-mediated transformation (ATMT) (Rho et al., 2001). The transformants with resistance to hygromycin B (40 μg/ml) were selected and screened by PCR with the primers ATMT-F/R and ATMT2-F/R to verify deletion by replacement. To complement the deletion strain, a fragment containing *TtTRI3* gene locus with its native promoter and terminator region was amplified by primers TRI3comp-F/R and ligated into a modified pCAMBIA1300 vector in which the original hygromycin B resistance gene was replaced with a G418-resistant gene. The final complementation vector was introduced in the mutant by ATMT and randomly inserted into the genome.

### Nucleic Acid Extraction and qRT-PCR

Vegetative mycelia were harvested from 5-day-old culture in a 250-ml flask containing 100 ml of liquid potato dextrose broth (PDB) incubated at 25°C with shaking (200 rpm). Genomic DNA and total RNA were extracted by the cetyl trimethylammonium bromide (CTAB) method (Del Sal et al., 1989) and the TRIzol reagent (TaKaRa), respectively. qRT-PCR was performed using SYBR Premix ExTaq (TaKaRa) on a Mastercycler Ep realplex thermo cycler (Eppendorf, Hamburg, Germany). Actin gene was chosen as the normalizing gene, and the expression levels of *TRI* genes were calculated by the 2^–ΔΔ*Ct*^ method (Livak and Schmittgen, 2001). Each reaction was repeated three times independently.

### Extraction and Analysis of Trichodermin

The wild-type ZJUF0986 and the mutant Δ*TtTRI3* were cultured in 150 ml of PDB medium in 500-ml Erlenmeyer flasks at 25°C in the dark. After 7 days’ incubation with shaking (200 rpm), the fermentation broth was filtered through three layers of lens paper. The filtered medium was extracted three times with equal volumes of ethyl acetate. The extract was dried by evaporation and dissolved in methanol and then analyzed by gas chromatography (GC)–MS as described by Cardoza et al. (2011).

### Antifungal Activity Test

The fermentation broth of *T. taxi* strains was harvested from 7-day-old cultures grown at conditions described above. *R. solani* was incubated on PDA containing 10 or 20% (v/v) harvested fermentation broth, using PDA containing 10 or 20% of non-inoculated fermentation broth as a reference set. Growth of *R. solani* on plates was monitored and recorded after 3 days’ incubation.

### Bacterial Cloning and Expression of *TtTRI3* Gene and Enrichment of the Protein Product

Reverse transcription and subsequent PCR was carried out with the PrimeScript^TM^ RT reagent Kit with gDNA Eraser (TaKaRa) and the primers TRI3cDNA-F/R. Purified PCR product was double-digested by *Bam*HI and *Hin*dIII and ligated into pQE30 (Qiagen) (Figure 6A). The resulted plasmid pQE30-TRI3 was sequencing verified and introduced into *E. coli* M15 (Qiagen), leading to strain M15-TRI3. Culture of M15-TRI3 was initiated and allowed to grow to OD_600_ at 0.6 with aeration at 37°C; 1 mM of IPTG was then added, and the culture was incubated overnight at 25°C with shaking. Cells were harvested by centrifugation at 10,000 *g* for 5 min at 4°C, re-suspended in buffer (pH 7.5) containing 50 mM of Tris, 200 mM of NaCl, 1 mM of β-mercaptoethanol, and 5 mM of imidazole. The cell suspension was subjected to sonication for a total of 15 min on ice, with 40% duty cycle (2-s working and 3-s pause). The cell lysate was clarified at 20,000 *g*, and cell debris was discarded. The His-tagged *Tt*TRI3 protein in the lysate was enriched using Ni-NTA beads (Invitrogen, Carlsbad, CA, United States) as recommended in the suppliers’ manual and concentrated and buffer-changed to 0.1 M of potassium phosphate buffer (pH 8.0) by centrifugation using Amicon^®^ Ultra centrifugal filters (Millipore, Billerica, MA, United States). Protein concentration was determined by the NanoDrop^TM^ 2000 UV-Vis spectrophotometer (Thermo Fisher Scientific, Waltham, MA, United States).

### Enzymatic Activity Assay of Acetyltransferase Activity of *Tt*TRI3

The activity of the acetyltransferase assay was performed according to Garvey et al. (2009) with slight modifications. Briefly, reaction mixtures were prepared at room temperature by combining 150 μl of 1.5 mM acetyl-CoA (Sigma-Aldrich Corp., St. Louis, MO, United States) with 50 μl of 4 mM trichodermol and 2 μl of 5 mg/ml bovine serum albumin (BSA). All components were dissolved in 0.1 M of potassium phosphate buffer (pH 8.0). The reaction was initiated by the addition of 10 μl of 290 μg/ml enriched enzyme described above. After incubation at 25°C for 5 h, the reaction mixture was extracted with an equal volume of ethyl acetate, and the extract was subjected to GC-MS analysis to verify the formation of trichodermin.

### Primer Sequences and Deposition of Nucleotide Sequences

All sequences of the primers used in the study are listed in Table 1. The nucleotide sequence of the contig containing *TRI* genes was directly submitted to GenBank under the accession number KY670722. The coding sequences of the individual *TRI* genes were also deposited with the following assigned accession numbers: KY860616 (*TtTRI3*), KY860617 (*TtTRI4*), KY860618 (*TtTRI6*), KY860619 (*TtTRI10*), KY860620 (*TtTRI11*), KY860621 (*TtTRI12*), and KY860622 (*TtTRI14*).

**TABLE 1:** Primers used in this study.

**Primer name**	**Sequence**	**Description**
TRI3-upF	TAGATTCGGAATAGTGGGTTGT	To amplify upstream arm flanking *TtTRI3*
TRI3-upR	CATTCATTGTTGACCTCCACTAAAA CATGGGCAGTGTTGGTAC	
TRI3-downF	GGGCAAAGGAATAGAGTAGATGAAC CTGTTTGCCCAATCCTTA	To amplify downstream arm flanking *TtTRI3*
TRI3-downR	CTCGTCATTCGCGTCCTACT	
HPH-F	TAGTGGAGGTCAACAATGAATG	To amplify the hygromycin B resistance cassette
HPH-R	CATCTACTCTATTCCTTTGCCC	
TRI3-NestF	ATGTCGACTCGGATGCTCATGGATA AAG	To obtain the final product from double-jointed PCR
TRI3-NestR	CGTCTAGATTCACAAGGCAACGTAA ACT	
ATMT1-F	GGCTCCGAGGCTATTGTG	To confirm insertion of the hygromycin B resistance cassette in *TtTRI3*
ATMT1-R	CGGGCGAAGGATTAAGAT	
ATMT2-F	GATTTCGGCTCCAACAAT	
ATMT2-R	TCGCCACAAACCTCAGTA	
TRI3comp-F	CGGTACCCGGGGATCCTTAAAGAA AGGGCAGGAG	To amply *TtTRI3* with its native promoter and terminator
TRI3comp-R	GCAGGTCGACTCTAGAGCATTTGC GAATAGTGAT	
TRI3cDNA-F	TG *GGATCC* AAACTTCCTGAGCTCCCAAAAT	For *TtTRI3* cDNA cloning
TRI3cDNA-R	TGGG *AAGCT T*CATTGGGAGAAGATTAACAT ATAGCTG	
ACTIN-F	GTTCTGTCCCTGTACGCTTC	qRT-PCR primers for actin gene
ACTIN-R	TAAGATCACGACCAGCCATG	
TRI3RT-F	CGATAACTTGGTGCGTTTGTC	qRT-PCR primers for *TtTRI3*
TRI3RT-R	TTGGATGCTTGGGTAGAATAGG	
TRI4RT-F	TTGTCAGGGATGGGTTTCAG	qRT-PCR primers for *TtTRI4*
TRI4RT-R	CCTCTCGCATTATCAGAAGCTC	
TRI6RT-F	AGCAGTGTAATAGTTGTAGTCCG	qRT-PCR primers for *TtTRI6*
TRI6RT-R	ACTATGAAGATTCGCCAACCC	
TRI10RT-F	GCCCATCGTCACCATTTATG	qRT-PCR primers for *TtTRI10*
TRI10RT-R	GCTCTTCCCGTTTCCATTTATC	
TRI11RT-F	AGCCATGAGAACCTTTAGCAG	qRT-PCR primers for *TtTRI11*
TRI11RT-R	ATTTGGAGAGTCGTTTGGAGG	
TRI12RT-F	CAGCAATAGAGGATTCAGGGAG	qRT-PCR primers for *TtTRI12*
TRI12RT-R	GTGGATCAATTTTCACCGCTG	

## Results

### Identification and Sequence Analysis of *TRI* Genes in *Trichoderma taxi*

**FIGURE 1:**
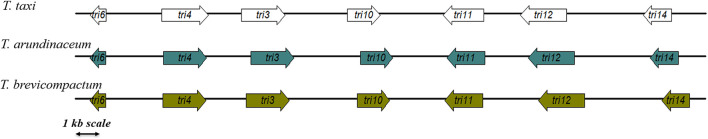
The *TRI* gene cluster in *Trichoderma taxi* ZJUF0986, *Trichoderma brevicompactum*, and *Trichoderma arundinaceum*. The orientations of arrows represented the direction of transcription, and the relative position of genes was proportionally scaled.

In order to understand the underlying biosynthetic pathway of trichodermin in *T. taxi*, we made use of the recently sequenced genome of *T. taxi* (manuscript in preparation). With the use of *T. arundinaceum TRI3* gene as a query to search against the genome, BLASTn identified the *TtTRI3*-containing contig as a 42.5-kb-long assembly (Figure 1). Further BLASTp search found that the contig encodes several other *TRI* orthologues, namely, *TtTRI4*, *TtTRI6*, *TtTRI10*, *TtTRI11*, *TtTRI12*, and *TtTRI14*, and that they were located in immediate vicinity on the contig. Along with *TtTRI3*, they appeared in the same order and orientation as those in *T. arundinaceum* and *T. brevicompactum* (Figure 1). Notably, the size of the genomic region between *TRI11* and *TRI12* in *T. taxi* is more like in *T. arundinaceum* than in *T. brevicompactum*, where the size is much larger (Figure 1). Consistent with previous observation in *Trichoderma* (Cardoza et al., 2011), *TRI5* is absent from the core *TRI* cluster in *T. taxi*. It is noteworthy that we are unable to recover the complete *TtTRI5* gene sequence from the contigs since querying *TaTRI5* against the assembled genome resulted in three separated short contigs (data not shown).

As expectable from the close phylogenetic relationship between *T. taxi* and *T. brevicompactum*/*T. arundinaceum* (Jaklitsch and Voglmayr, 2015), the proteins encoded by the *TRI* cluster display a high degree of identity, suggesting that they are structurally and functionally conserved (Table 2). Results from protein domain search identified *Tt*TRI3 as a TRI3 family 15-*O*-acetyltransferase, *Tt*TRI4 and *Tt*TRI11 as cytochrome P450 domain-containing oxygenases, *Tt*TRI12 as a fungal trichothecene efflux pump, *Tt*TRI6 as a C_2_H_2_-type zinc finger domain-containing transcriptional regulator, and *Tt*TRI10 likely to be a transcription factor. No match in protein domain database was found for *Tt*TRI14. Though orthologues of TRI14 have been found in other Hypocreales, such as *Fusarium* spp. (Peplow et al., 2003), *Stachybotrys* spp., and *Cordyceps* spp., the function of the protein is currently unknown.

**TABLE 2:** Comparison of *TRI* genes from *Trichoderma taxi* with orthologues from *Trichoderma arundinaceum*, *Trichoderma brevicompactum*, and *Fusarium sporotrichioides*.

***T. taxi TRI* genes**	**Protein size (aa)**	**Protein domain**	**Protein identities (%)**		
			***T. arundinaceum***	***T. brevicompactum***	***F. sporotrichioides***
*TRI3*	519	15-*O*-Acetyltransferase TRI3 family	83	83	48
*TRI4*	517	Cytochrome P450 domain	92	94	73
*TRI6*	218	C_2_H_2_-type zinc finger domain	96	95	51
*TRI10*	422	Fungal trans 2 (PF11951)	97	97	56
*TRI11*	498	Cytochrome P450 domain	92	92	39
*TRI12*	595	Fungal trichothecene efflux pump	86	91	57
*TRI14*	367	NF^a^	95	94	64

*^*a*^NF, not found.*

### Disruption of *TtTRI3* Impaired Production of Trichodermin and Antagonism Against *Rhizoctonia solani*

**FIGURE 2:**
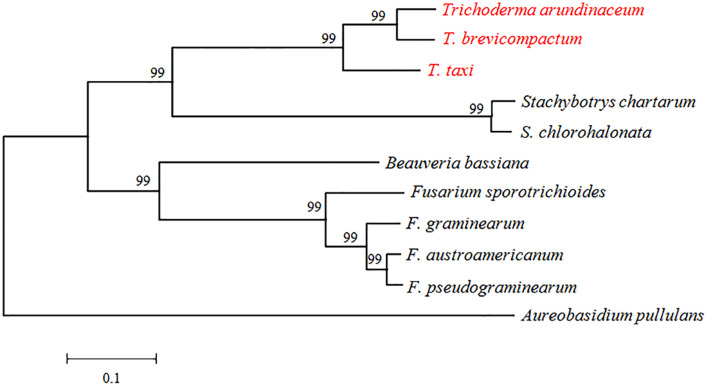
Phylogenetic tree based on alignment of *TRI3* from *Trichoderma*, *Stachybotrys*, *Beauveria*, *Fusarium*, and *Aureobasidium*. The species were chosen as representatives of hits from a BLASTp search. Sequence alignment and tree building were performed by MEGA5.0 using the neighbor-joining method, and phylogeny was tested by 500 bootstrap replications. The numbers on branches were calculated bootstrap values. The accession numbers for *TRI3* genes were as follows: *TaTRI3* (CAY87361), *TbTRI3* (CBY93780), *Stachybotrys chartarum TRI3* (KEY64037), *Stachybotrys chlorohalonata TRI3* (KFA68878), *FgTRI3* (BAC22114), *FpTRI3* (XP_009263555), *FaTRI3* (AAM48923), *FsTRI3* (AAK33072), *BbTRI3* (XP_008602023), and *ApTRI3* (KEQ86789).

**FIGURE 3:**
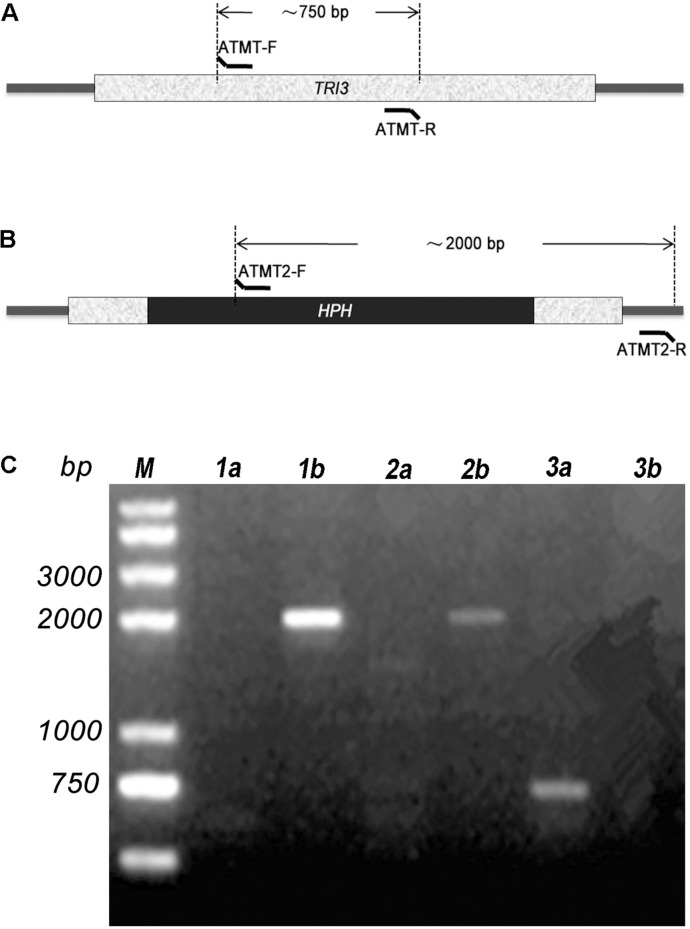
PCR confirmation of insertion of the *HPH* cassette in *TRI3* gene. The relative positions of primers ATMT-F/R **(A)** and ATMT2-F/R **(B)** are shown. **(C)** Two transformants were confirmed by PCR. Primer pair ATMT-F/R was used for lane 1a, 2a, and 3a; while ATMT2-F/R was used for lane 1b, 2b, and 3b. 1a, 1b and 2a, 2b were designated for the two transformants. Wild-type strain was used in 3a and 3b.

*TRI3* had not been characterized in *Trichoderma* spp. when we initiated this study. It is highly conserved in the genus *Trichoderma* and other Sordariomycetes, but the distance (i.e., branch length) between *Trichoderma* TRI3 and other fungi is high, suggesting potential differences in function (Figure 2). In *Fusarium*, TRI3 catalyzes the acetylation of the hydroxy group at C-15 of the trichothecene skeleton (McCormick et al., 1996). In *T. brevicompactum*, TRI3 was proposed to be responsible for the acetylation of the hydroxy group at C-4 of the trichothecene skeleton (Cardoza et al., 2011), but this hypothesis has not been experimentally tested yet. We therefore generated a *TtTRI3* null mutant by replacing the *TRI3* ORF by a 1.4-kb *HPH* cassette. Successful gene replacement was confirmed by PCR screening (Figure 3).

**FIGURE 4:**
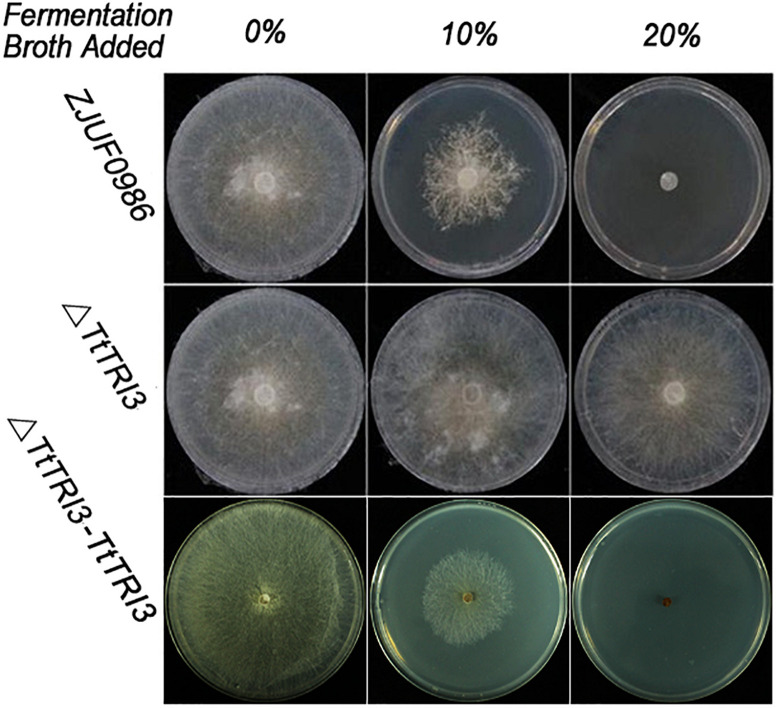
Disruption of *TRI3* relieved growth inhibition to *Rhizoctonia solani*. From left to right: 0% (v/v), 10% (v/v), or 20% (v/v) of harvested fermentation broth from wild-type strain or *TtTRI3* disruption strain, or complementation strain was added in potato dextrose agar (PDA). Photos were taken 3 days after incubation.

There were no significant phenotypic changes observed in colonial morphology and growth rate between wild type and Δ*TtTRI3* (data not shown). However, in contrast to the wild type, Δ*TtTRI3* lost the ability to secrete molecules that inhibited growth of *R. solani* (Figure 4): when 10% of the filtered fermentation broth from the parent strain was added to a PDA plate, growth of *R. solani* on the plate was strongly inhibited, and complete inhibition was observed by addition of 20%. In contrast, even adding 20% of the fermentation broth of the knockout strain Δ*TtTRI3* to the plate did not impair growth of *R. solani*. This suggests that TRI3 catalyzes an essential step in the generation of the compound that inhibits *R. solani* growth.

**FIGURE 5:**
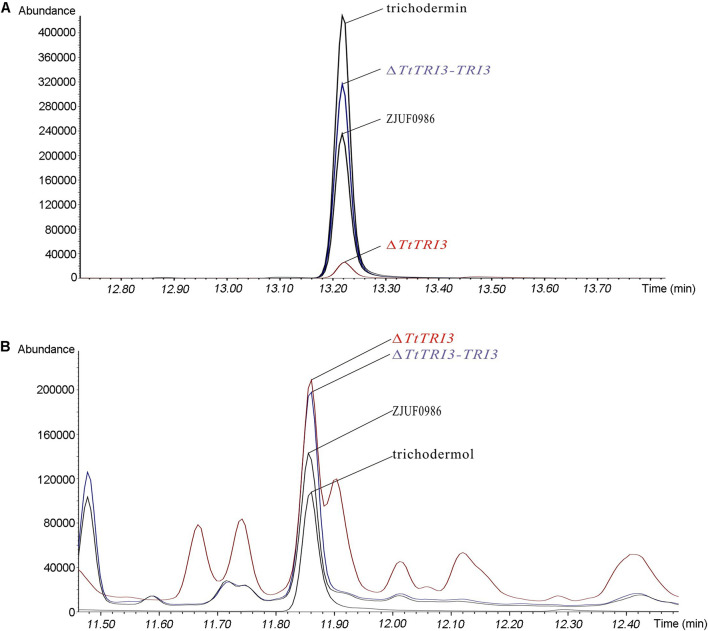
Trichodermin and trichodermol production in strain ZJUF0986, Δ*TtTRI3*, and Δ*TtTRI3-TRI3*. Amount of trichodermin **(A)** and trichodermol **(B)** in fermentation broth was detected by gas chromatography (GC)–MS. Peaks for trichodermin and trichodermol were determined by the corresponding reference chemicals. The concentration of reference chemicals in the prepared samples was 188 ppm (part per million) for trichodermin and 42 ppm for trichodermol.

To test whether deletion of *TtTRI3* indeed affects the production of trichodermin, the fermentation broth from both the wild-type culture and the Δ*TtTRI3* culture was subject to GC-MS analysis. This showed that the production of trichodermin was significantly decreased in the *TtTRI3* strains (Figure 5A). Interestingly, the concentration of trichodermol, the immediate trichodermin precursor, did not accumulate to higher levels in the Δ*TtTRI3* strain and remained at the same level as in the wild-type strain (Figure 5B), suggesting the operation of an effective feedback control in its biosynthesis. These data demonstrate that TRI3 plays an essential role in the conversion of trichodermol to trichodermin and that trichodermin confers *T. taxi* most (if not all) of its ability to inhibit growth of *R. solani*.

To further confirm that *TRI3* is responsible for converting trichodermol to trichodermin in *T. taxi* and that trichodermin is the main component conferring inhibitory effect to *R. solani*, wild-type *TtTRI3* gene with its native promoter and terminator was introduced into the Δ*TtTRI3* mutant, resulting in the complementation strain Δ*TtTRI3*–*TRI3*.

The *TRI3* complementation restored the knockout strain’s ability to inhibit growth of *R. solani* to that of the wild-type level; i.e., 10% of added fermentation broth largely inhibited growth of *R. solani* and 20% resulted in complete inhibition (Figure 4). In addition, GC-MS analysis of the fermentation broth from Δ*TtTRI3*–*TRI3* showed that trichodermin biosynthesis was recovered and that its abundance in the complementation strain was even higher than that in the wild-type strain (Figure 5A). These data confirmed that the results obtained with the *TtTRI3* knockout strain are valid, and TRI3 plays an essential role in converting trichodermol into trichodermin in *T. taxi*.

### *Tt*TRI3 Converts Trichodermol to Trichodermin

**FIGURE 6:**
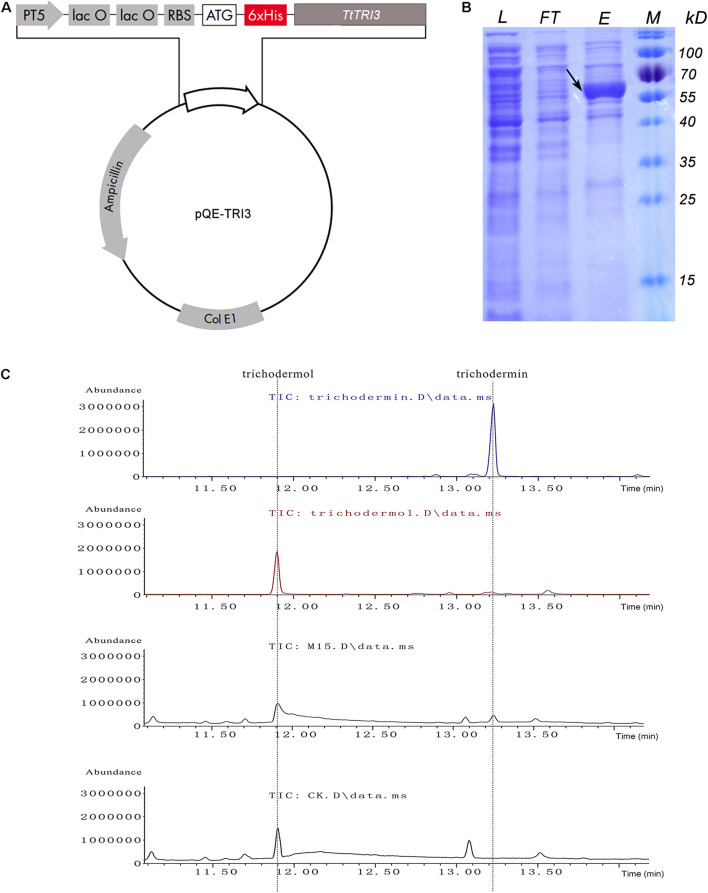
*In vitro* conversion of trichodermol to trichodermin catalyzed by bacterial expressed *Tt*TRI3. **(A)** The structure of the constructed plasmid for expression of *Tt*TRI3. The 6xHis tag was engineered to fuse immediately upstream of the *Tt*TRI3 coding region. **(B)** Sodium dodecyl sulfate–polyacrylamide gel electrophoresis (SDS-PAGE) shows the purification of *Escherichia coli*-expressed *Tt*TRI3. The black arrow indicates the band of the target protein. The molecular weight (MW) of this band on the gel is consistent with the calculated theoretical MW, 60 kDa. L: cell lysate; FT: flowthrough from Ni-NTA column after cell lysate was loaded; E: eluate from *Tt*TRI3-bound column; M: protein marker. **(C)** GC-MS analysis results showed that addition of *Tt*TRI3 resulted in the appearance of trichodermin in reaction mixture. The upper two curves show the respective retention time of trichodermin and trichodermol. The two curves at the bottom represent curves obtained from ethyl acetate extract from trichodermol reaction mixture, with combination of either *Tt*TRI3 (sample M15) or cell lysate prepared from *Escherichia coli* cell bearing empty vector pQE30 (sample CK).

To examine the potential conversion of trichodermol to trichodermin by *Tt*TRI3 also *in vitro*, *TtTRI3* was recombinantly produced with a His-tag in *E. coli* and purified to >70% homogeneity (estimated by ImageJ: https://cnij.imjoy.io/) by Ni-NTA resin batch treatment (Figure 6B). A biochemical assay combining trichodermol, *Tt*TRI3, and acetyl-CoA was established to test if this enriched *Tt*TRI3 was able to catalyze acetylation of trichodermol *in vitro*. GC-MS analysis of the ethyl acetate extract of the reaction mixture showed that a new peak appeared at the position of trichodermin, while the peak was missing when the cell lysate was prepared from *E. coli* cells where the empty vector was introduced instead (Figure 6C). Exclusion of acetyl-CoA or the recombinantly produced *Tt*TRI3 protein from the mixture also prevented the formation of trichodermin (data not shown), thus proving that TRI3 and acetyl-CoA are required for the reaction to happen.

### Disruption of *TtTRI3* Gene Downregulated the Expression of Other *TRI* Genes

**FIGURE 7:**
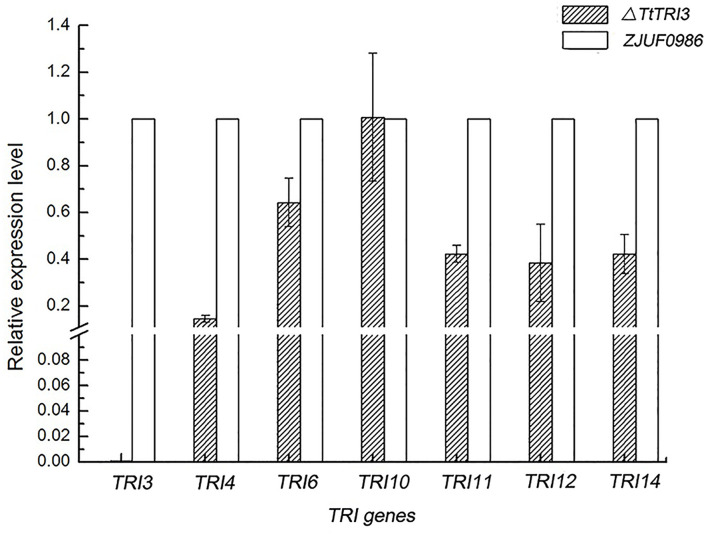
Comparison of *TRI* genes expression level between wild-type strain and Δ*TtTRI3*. Expression level for *TRI* genes was determined by qRT-PCR. Expression level of genes in ZJUF0986 was normalized as 1. Calculation was based on data obtained from three independent experiments.

We have noted above that the deletion of *TtTRI3* did not result in significant over-accumulation of trichodermol, suggesting a tight feedback control. To test whether this would involve regulation of gene expression, we therefore quantified the expression level of *TtTRI4*, *TtTRI6*, *TtTRI10*, *TtTRI11*, and *TtTRI12* in the parent strain ZJU0986 and its knockout mutant Δ*TtTRI3*. As illustrated in Figure 7, we indeed found that the expression of all structural genes was decreased in the mutant by more than 50%, whereas the expression of the regulator genes *TtTRI10* was unaffected and that of *TtTRI6* reduced by only 30%. These data suggest the operation of a feedback loop in the trichodermin biosynthesis pathway in *T. taxi*.

## Discussion

**FIGURE 8:**
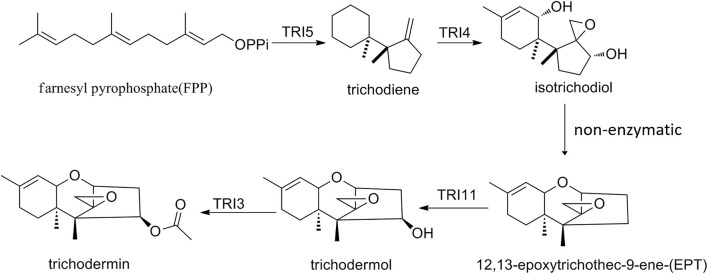
The proposed trichodermin biosynthetic pathway in *Trichoderma taxi*.

Trichodermin can be produced by *T. taxi*, *T. brevicompactum*, and *T. arundinaceum* (Nielsen et al., 2005; Zhang et al., 2007). Since its discovery, this fungal secondary metabolite has been shown to have several important bioactivities such as growth inhibition to fungal pathogens, cytotoxicity, and recently anticancer activity (Godtfredsen and Vangedal, 1965; Wei et al., 1974; Su et al., 2013; Chien et al., 2016; Uchida et al., 2016). Although chemical synthetic routes for trichodermin had been developed not long after its discovery (Colvin et al., 1973), it was not until recently that the biosynthetic pathway was elucidated (Cardoza et al., 2011). Like other trichothecenes, biosynthesis of trichodermin starts from the cyclization of FPP to form trichodiene by the TRI5 trichodiene synthase. TRI4 then oxidizes trichodiene to isotrichodiol, which subsequently forms EPT by non-enzymatic dehydration. TRI11 then hydroxylates EPT to trichodermol (Figure 8), and TRI3 was proposed to catalyze the acetylation of trichodermol to trichodermin (Cardoza et al., 2011). Recently, TRI3 has been shown to be involved in trichodermin synthesis in an endophytic *T. brevicompactum* by mutational analysis (Shentu et al., 2018). In this study, for the first time, we present direct evidence that *Tt*TRI3 is capable of performing the acetylation of trichodermol to trichodermin, using acetyl-CoA as acetyl donor. Our results also imply that biosynthetic pathway of trichodermin is conserved in *Trichoderma* species capable of producing it.

Some *Trichoderma* species such as *T. brevicompactum* were reported to produce both trichodermin and harzianum A (HA), depending on the strains and the cultivation conditions (Nielsen et al., 2005). HA differs from trichodermin in that it is esterified at C-4 with octa-2*Z*,4*E*,6*E*-trienedioic acid while trichodermin is esterified with acetic acid. In HA-producing strains, octa-2*Z*,4*E*,6*E*-trienedioic acid was also detected, suggesting that HA is synthesized in one single step from trichodermol and an octa-2*Z*,4*E*,6*E*-trienedioic group donor (Nielsen et al., 2005), which has been proved in *T. arundinaceum* by gene disruption (Proctor et al., 2018). Here, we showed that acetylation of trichodermol is also catalyzed by TRI3.

In *Fusarium*, TRI3 functions to acetylate the hydroxyl group at C-15 of the trichothecene skeleton (McCormick et al., 1996). Gene *TRI3* in *F. sporotrichioides* (*FsTRI3*) shares 48% identity in translated amino acid sequence with TRI3 from *Trichoderma* (Table 2; Cardoza et al., 2011), which is rather low but consistent with a functional difference (shift of acetylation site from C-15 in *Fusarium* to C-4 in *Trichoderma*) between the two TRI3 enzymes. Intriguingly, a similar situation has been observed in the case of *TRI11*/TRI11, where amino acid sequence identity between the two genera is 39%, much lower than that of other TRI proteins, such as TRI4 (73%), TRI5 (57%), and TRI12 (55%) (Table 2; Cardoza et al., 2011), consistent with the fact that *Fusarium* TRI11 oxygenates trichothecene skeleton at C-15, while *Trichoderma* TRI11 does so at C-4 position and that other TRI proteins are functionally more conserved (Alexander et al., 1998; Cardoza et al., 2011). It is reasonable that the two genes are co-evolved since the substrate upon which TRI3 acts depends on TRI11.

In this study, the disruption of *TtTRI3* gene severely reduced the expression of other trichodermin biosynthesis genes, while the regulatory gene *TtTRI6* was less and *TtTRI10* not at all affected. The results were in accordance with the observation that deletion or silencing of *TaTRI4* in *T. arundinaceum* reduced expression level of *TaTRI5* (Malmierca et al., 2012). Given the sampling time points in our study, our results are also in good compliance with those obtained by Shentu et al. (2018) where *TRI* genes were downregulated at mid–late growth phase. Surprisingly, the disruption of *TaTRI5* in the HA biosynthetic pathway upregulated all other *TaTRI* genes except *TaTRI4* (Malmierca et al., 2013). Our data suggest that the expression of *TRI* genes is subjected to metabolite feedback repression, a common phenomenon in biosynthetic pathways (Vasanthakumar et al., 2013; Pratelli and Pilot, 2014; Ness, 2015), likely by TRI10. In *T. taxi*, a decrease in the concentration of trichodermin may elicit this regulation, because the concentration of trichodermol was not significantly altered in the knockout mutant. However, other metabolites of the biosynthetic pathway, which have not been measured, may also be involved. In *Fusarium*, *TRI6* and *TRI10* function as regulatory genes to control expression of other genes in trichothecene biosynthesis (Proctor et al., 1995; Tag et al., 2001; Nasmith et al., 2011). *Fs*TRI6 binds to the consensus sequence TNAGGCCT that is found in the promoter of most *TRI* genes (Hohn et al., 1999). The disruption of *Fusarium TRI6* greatly reduced the expression of *TRI5* and *TRI4*, and the mutant failed to accumulate T-2 toxin (Proctor et al., 1995). *Fs*Tri10 was also found to regulate all other *TRI* genes and several putative isoprenoid biosynthetic genes (Peplow et al., 2003). How TRI6 and TRI10 cooperate to regulate the expression of other *TRI* genes in *Trichoderma*, possibly in coordination with metabolite feedback, would be an interesting topic for the future.

## Data Availability Statement

The datasets presented in this study can be found in online repositories. The names of the repository/repositories and accession number(s) can be found in the article/supplementary material.

## Author Contributions

HC performed TRI3 expression, purification, and the *in vitro* reaction and prepared the original manuscript. LM carried out all the GC-MS experiments and analyzed the data. NZ sequenced the genome and constructed the mutant strains. CX performed the antifungal activity test. JL and CZ designed and supervised the research and revised the manuscript. WW assisted with chemical structures and manuscript revision. SX and CK perfected the language and revised the manuscript. All authors discussed, edited, and approved the final version.

## Conflict of Interest

HC and JL was employed by China Tobacco Guizhou Industrial Co., Ltd. The remaining authors declare that the research was conducted in the absence of any commercial or financial relationships that could be construed as a potential conflict of interest.

## Publisher’s Note

All claims expressed in this article are solely those of the authors and do not necessarily represent those of their affiliated organizations, or those of the publisher, the editors and the reviewers. Any product that may be evaluated in this article, or claim that may be made by its manufacturer, is not guaranteed or endorsed by the publisher.

## Footnotes

1
http://blast.ncbi.nlm.nih.gov/Blast.cgi

2
http://www.acaclone.com/

3
http://pfam.sanger.ac.uk/
